# Retraction: Amyand Hernia With Acute Appendicitis: A Rare Type of Hernia

**DOI:** 10.7759/cureus.r88

**Published:** 2024-01-25

**Authors:** Majd A Assad, Thamer A Boushal, Zeyad W Halawani, Rayan S Alruwaili, Yakeen H Alkuwaiti, Aisha M Alghamdi, Wedad A Bahri, Bader M Albilasi, Shrouq M Alghamdi, Bushra M Alghanmi, Manal M Alghanmi, Abdulaziz J Alghamdi, Moath M Alkhaldi, Malak Alshammari

**Affiliations:** 1 Surgery, College of Medicine, Imam Abdulrahman Bin Faisal University, Dammam, SAU; 2 Surgery, College of Medicine, King Faisal University, Al-Ahsa, SAU; 3 Surgery, College of Medicine & Medical Science, Arabian Gulf University, Manama, BHR; 4 Surgery, College of Medicine, Al-Baha University, Al-Baha, SAU; 5 Surgery, College of Medicine, Batterjee Medical College, Jeddah, SAU; 6 Surgery, College of Medicine, Jouf University, Al-Jawf, SAU; 7 Surgery, College of Medicine, Umm Al-Qura University, Mecca, SAU; 8 Surgery, College of Medicine, Ibn Sina National College for Medical Studies, Jeddah, SAU; 9 Surgery, College of Medicine, Jazan University, Jizan, SAU; 10 Internal Medicine, College of Medicine, Imam Abdulrahman Bin Faisal University, Dammam, SAU

The Editors-in-Chief have retracted this article. Concerns were raised regarding the identity of the authors on this article. Specifically, Faisal Alhaway and Malak Shammari have stated that they were added to this article without their knowledge or approval. The identity of the other authors could also not be verified. As the appropriate authorship of this work cannot be established, the Editors-in-Chief no longer have confidence in the results and conclusions of this article.

